# Practice recommendations and referrals, perceptions of efficacy and risk, and self-rated knowledge regarding complementary medicine: a survey of Australian psychologists

**DOI:** 10.1186/s12906-023-04288-y

**Published:** 2024-01-02

**Authors:** Carrie Thomson-Casey, Erica McIntyre, Kris Rogers, Jon Adams

**Affiliations:** 1 School of Public Health, Faculty of Health, University of Technology Sydney, Sydney, Australia; 2 Faculty of Health, Southern Cross University, Gold Coast, Australia; 3 Institute for Sustainable Futures, University of Technology Sydney, Sydney, Australia

**Keywords:** Psychology practice, Clinical practice, Complementary medicine

## Abstract

**Background:**

Many people with mental health problems use a range of complementary medicine (CM), including over the counter products, practices, and utilise the services of CM practitioners. Psychologists are likely to consult with clients using CM, in some form, as part of their broader mental health care. The aim of this research was to determine the number of types of CM products, practices, and practitioners are recommended and/or referred by Australian psychologists as part of their clinical practice, as well as explore the relationship between psychologists’ perspectives on the risk and relevance of engaging with CM in psychology.

**Methods:**

Survey data was collected from psychologists in clinical practice who self-selected to participate in the study via an online 79-item questionnaire exploring core aspects of CM engagement in psychology clinical practice.

**Results:**

Amongst the 201 psychologists, 5% reported not recommending any type of CM, with 63% recommending four or more types of CM. Further, 25% had not referred to a CM practitioner, while 33% had referred to four or more types of CM practitioner. Psychologists are recommending and referring to CM even when they perceive their knowledge of CM to be poor, and that engaging with CM was a risk.

**Conclusion:**

This study provides insights into psychologist perceptions of CM within psychology practice and how these perceptions are associated with rates of recommending and referring to CM as part of their clinical practice. These findings may inform the development of CM relevant education and guidelines for psychologists.

**Supplementary Information:**

The online version contains supplementary material available at 10.1186/s12906-023-04288-y.

## Background

The use of complementary medicine (CM)—a range of practices, products and systems of care not traditionally associated with the conventional medical profession or curriculum [4]—has gained increasing acceptance alongside conventional medical treatments in a number of health settings [1, 10, 19, 23, 43, 46, 56]. Amongst these developments, some psychologists have reported positive views toward the use of CM as part of, or as an accompaniment to, the mental health care they provide [27, 28, 48].

Some CM approaches such as nutrition, movement therapies, and massage have been identified as effective for addressing certain mental health symptoms [9, 18, 20, 40, 45, 57] and some CM approaches have become more widely accepted amongst psychologists (e.g., eye movement desensitisation and reprocessing, guided imagery, meditation, mindfulness) and integrated into their contemporary practice [21, 30, 35, 39]. Many diagnosed with mental health problems are CM users [12, 14, 32, 33, 47, 54] and a psychologist is likely to consult with some clients who use CM as part of their wider mental health care. For example, a study of Turkish people with mental health problems found 62.2% had used CM in some form in the last 12 months [7]. Similarly, a study of Australian adults diagnosed with mental health problems reported 42.4% consulted a CM practitioner, 56.9% used a CM product, and 23% used a CM practice in the previous three years [33].

The *integration* of CM within psychology practice (as within other areas of health practice) can take a number of forms [3] including through direct *discussion* about CM with a client in consultation (e.g., discussing potential herb-drug interactions), *recommending* CM to a client (e.g., suggesting the client attend a yoga class for relaxation) and *referring* a client to a CM practitioner (e.g., verbal or written referral to a naturopath to explore evidence based herbal interventions for anxiety). Integration may also be via *direct practice application* of CM to the client by a psychologist (e.g., explicit instructions regarding a client’s nutritional intake/diet in the context of evidence-based nutritional psychiatry).

The safe integration of CM into mental health care is not without challenges. Mental health practitioners, including psychologists, often report gaps in their knowledge regarding relevant evidence-based CM [8, 11, 36, 44, 52] associated with an inability to discuss CM with clients or to recommend or facilitate referral to CM if requested by a client [27, 31, 38]. Further, some psychologists engaging with CM in their practice complain of a lack of explicit policy and guidelines for the safe integration of CM into psychology practice [22, 24, 27]. Analysis of guidelines from Australian psychology professional associations reveals limited mention of CM, nor how psychologists could safely integrate CM into their practice [51].

Unfortunately, much detail and many issues around CM integration by psychologists remain unexplored including what influences psychologists to engage, or not engage, with types of CM in their clinical practice. Further, we still know little regarding Australian psychologists’ perspectives about the efficacy, risks and relevance of CM in psychology. The study reported here has been designed to directly address these important research gaps.

## Methods

### Aims and objective

The aim of this research was to determine how many types of CM products, CM practices, and CM practitioners are recommended and/or referred by Australian psychologists as part of their clinical practice as well as explore the relationship between psychologists’ perspectives about the efficacy, risks and relevance of CM in psychology, and their self-rated knowledge of CM, with the number of types of CM to which psychologists are recommending or referring their clients.

### Study design

This study employed an online survey administered exclusively to Australian psychologists who were fully registered and working in a clinical practice setting at the time of survey between February and April of 2021. Email invitations to participate in the study were sent to psychologists whose contact details were collected from their publicly available websites. The recruitment emails contained information about the study, consent forms, and a link to complete the survey online. All participants were sent a reminder email four weeks following the initial invitation email. An advertisement inviting psychologists to participate in the research was also placed on two psychology professional association websites (Australian Association of Psychologists Incorporated and the Australian Psychological Society) and on relevant social media sites including Twitter, LinkedIn and Facebook. Participants accessed the survey via an anonymous link (embedded in the email, website advert or in social media post), which initially directed the psychologist to the participant information and consent form followed by the link to the open survey using Qualtrics software, Version 2021 [42]. The information page at the beginning of the survey included project details such as ethical approval, data protection, and voluntary participation. The information page also served as the participant consent form. Participants indicated their written consent after reading the information and consent page and clicking on the button confirming their agreement to proceed with the survey. Participants who completed the online survey were invited to supply their email address to enter a prize draw to win a gift voucher to the value of $250. Ethical approval was attained from the University of Technology Sydney Human Research Ethics Committee [ETH20-5138].

### Sample

The survey was distributed to 1,479 Australian psychologists working in clinical practice at the time of recruitment. All psychologists (psychologists with general registration, and those psychologists with general registration plus an area of practice endorsement (AoPE)) were eligible to participate in the study. However, only responses from those psychologists working in an Australian clinical practice setting (e.g., inpatient hospital, private practice) and directly with clients were included in the data analysis for this study.

All psychologists in Australia (*n* = 34,872) are considered to hold general registration, which enables them to use the title of *psychologist* (Psychology Board of Australia, 2022). Some psychologists with additional tertiary training in psychology may also hold an AoPE enabling them to use a restricted title (e.g., clinical psychologist). These AoPE titles are clinical neuropsychologist, clinical psychologist, community psychologist, counselling psychologist, educational and developmental psychologist, forensic psychologist, health psychologist, organisational psychologist, and sport and exercise psychologist. A psychologist with an AoPE title has general registration *plus* an AoPE. To clarify, a psychologist with general registration *without* an AoPE (i.e., psychologist) can work in clinical practice settings. However, working in clinical practice does not mean a psychologist is a clinical psychologist.

Psychologists self-selected to participate by clicking on the survey link in the email invitation or on the websites listed above. An initial screening question asked participants if they were a psychologist who undertakes the work of a psychologist. Participants who selected “No” were redirected out of the survey. The current study focused exclusively on registered psychologists in clinical practice in Australia. The original sample size was planned to be 400, based achieving a 0.10 confidence interval width on estimates of prevalence of binary questionnaire items. As noted above, we were able to recruit 231 participants, of which 201 passed the inclusion criteria and were used in this study. With this sample size we are able to estimate a confidence interval for the prevalence of a single binary item with a CI width of 0.14; or compare a continuous or binary variable between two equally sized groups with 0.8 power and an effect size of 0.39 (Cohen’s d).

### Instrument

The construction of survey items was informed by previous literature on psychologist engagement with CM to produce survey items that best captured the ways psychologists might be engaging with CM, including the types of CM products and practices they had ever recommended and the types of CM practitioners to which they had ever referred. Participants were also provided with a definition of CM in line with the World Health Organisation [55]. The 79-item questionnaire aimed to examine psychologists’ perspectives relating to how CM is/is not relevant and/or appropriate to (their) psychology practice, their clients, and the treatment of mental health problems. Prior to recruitment, the survey was tested for face validity and functionality by three PhD students from psychology adjacent fields. As part of this process, any identified necessary changes were undertaken to provide clearer definitions and reduce repetitive questions. Based on survey testing feedback, the time required to complete the survey was approximately 15 min. Where relevant this paper adhered to the CHERRIES checklist for reporting results of internet e-surveys [15, 16]. The study reported in this paper focused on the survey data sections examining psychologist demographics, types of CM products and/or practices recommended, and/or CM practitioners referred to, psychologists’ perspectives on CM within psychology, as well as psychologist self-rated knowledge of CM types.

### Demographics

Demographic data included year of birth, gender identity, and the predominant state/territory in which they practice. Psychologists were also asked to provide practice characteristics, such as AoPE, their work setting (solo or group setting), and years in practice as a psychologist.

### Perspectives on CM within psychology

Participating psychologists were invited to rate their agreement with statements related to efficacy and relevance of CM to psychology and psychology practice. There were two efficacy questions rated on a six-point Likert scale (*strongly agree, agree, somewhat agree, somewhat disagree, disagree to strongly disagree*). Perspectives about the relevance of CM to psychology and psychology practice were addressed across thirteen statements (e.g., It is important for psychologists to understand and engage with their client’s preference for CM as part of their mental health treatment). The statements about the relevance of CM were also rated on a six-point Likert scale from strongly agree to strongly disagree. To reduce risk of bias there were six questions across the perspectives categories that described CM as a risk to psychology. For example, “CM is not a good match with psychology” and “Psychology integrating with CM puts psychology’s reputation at risk”. Psychologists were also invited to select types and frequency of CM products, practices and practitioners they had ever recommended and/or referred their clients to as part of their clinical practice.

### Self-rated knowledge of CM

This section asked psychologists to self-rate their knowledge across eleven types of CM: Aboriginal and Torres Strait Islander Traditional Medicine /Healing practices, acupuncture, dietary intervention, exercise/movement interventions, herbal medicine, hypnotherapy, massage, meditation, nutrition supplements, probiotic supplements, and yoga. Psychologists could rate their knowledge on a four-point Likert scale from excellent, good, fair, and poor.

### Data analysis

IBM SPSS Statistics Premium Edition Version 27 (Armonk, New York, IBM Corp) was used to analyse the data. Prior to conducting the analyses, raw survey data were screened for any missing or incomplete responses. During this process, nine cases were removed as the data (responses) were incomplete. After removal of the nine cases, 222 cases were included in the initial analyses which identified significant outliers. On review the outlier responses were mostly from cases who did not work in clinical practice settings. These cases were removed resulting in 201 participants in the final data set. Descriptive statistics were used to determine the percentages and frequencies. A Poisson regression model was used to estimate rate ratios between demographic and practice covariates and the outcomes of CM engagement l (number of types of *recommending* CM products and practices and *referring* to CM practitioner types, agreement with statements about risks and relevance of CM, self-rated knowledge of types of CM).

Variables and categories were created to best capture psychologist responses to survey items. For analysis the types of CM products and practices were categorised into six groups that were informed by previous literature (Ng et al., 2016; Wieland et al., 2011) and consistent with the definition of CM used in this study: mind/body approaches (hypnotherapy, meditation, yoga), movement approaches (exercise and movement-based activities, such as walking), ingestive therapies (herbal medicine, probiotics, vitamin and nutrition supplements), dietary changes and manual approaches (acupuncture and massage). The sixth category cultural and spiritual approaches included participant’s free text responses indicating recommending or referring to music, creative arts, prayer and spirituality, and Aboriginal and Torres Strait Islander traditional healing. The number of types of CM products and/or practices psychologists recommend to their client was collapsed into three categories: recommended none; recommended one to three types of CM products and/or practices; and recommended four plus types of CM products and/or practices.

Psychologists could also select from six categories of CM practitioners to which they have ever referred their clients. Practitioner types included mind/body practitioners (e.g., hypnotherapists and yoga teachers), movement practitioners (e.g., exercise and movement trainers and/or coaches), practitioners who predominantly prescribe ingestibles category (e.g., naturopaths, herbalists, and traditional Chinese practitioners), prescribes nutrition (e.g., nutritionists), manual practitioners (e.g., acupuncturists, massage therapists), and cultural/spiritual practitioners (e.g., Aboriginal and Torres Strait Islander traditional healers). The number of types of CM practitioner to which a psychologist referred their clients was combined into three categories: refers to none, refers to one to three types of CM practitioner, and referred to four plus types of CM practitioner.

Variables relating to perspectives about the efficacy and relevance of CM, and self-rated knowledge of CM, were also adjusted for the purpose of analysis. Due to the small number of responses in some categories the statement responses were collapsed into fewer categories. Both categories relating to agreement with perspectives about CM (efficacy and relevance) were collapsed into two responses of either agree or disagree. Finally, the self-rated knowledge category was also collapsed into two responses of either excellent/good or fair/poor.

Count variables were also created for the Poisson regression analyses. A variable representing the total number of types of CM products and practices that psychologists would refer patients to was created, and similarly one for the number of types of CM practitioners that psychologists refer to as part of their clinical practice. For the additional summary data tables, the count variables were converted to ordinal variables (for grouping into three categories) for both recommend or referred; none, one to three types, and four or more types of CM.

## Results

Of the total participants, 66% (*n* = 134) accessed the survey via email invitation link and the remaining 345% of participants (*n* = 68) accessed the survey via website or social media link. A large majority (77%) of people who accessed the survey completed it.

### Participant characteristics

The study sample (*N* = 202) was comprised of 165 women (81.6%), 36 men (17.8%) and one person who identified as other (0.5%). The mean age of psychologist participants was 48 years (*M* = 48.260, *SD* = 26.53). All of the Australian states and territories were represented within the sample, with highest representation from New South Wales (*n* = 65) and Queensland (*n* = 64), and the lowest from Northern Territory (*n* = 1). Most psychologists in the study identified as having the AoPE as a clinical psychologist (*n* = 79). Psychologists with general registration (*n* = 76) were also represented. There were also psychologists who identified as *Other AoPE* including counselling psychologists (*n* = 25), forensic psychologists (*n* = 8), health psychologists (*n* = 7), educational and developmental psychologist (*n* = 6) and one community psychologist. Solo private practice was the most common work setting reported among participants (*n* = 137). The highest proportion of participants had 11 to 20 years of clinical experience (*n* = 72) and the lowest proportion had 31 plus years of clinical experience (*n* = 31). Table 1 provides a summary of demographic and professional characteristics.

**Table 1:** Psychologist demographic and practice characteristics, by area of practice endorsement (AoPE)

	**All** **(*n* = 202)**		**General psychologist** **(*n* = 76)**		**Clinical psychologist** **(*n* = 79)**		**Other AoPE psychologist** **(*n* = 47)**	
	***n***	**%**	***n***	**%**	***n***	**%**	***n***	**%**
**Gender**								
*Female*	165	81.7	62	81.6	68	86.1	35	74.5
*Male*	36	17.8	14	18.4	10	12.7	12	25.5
*Other*	1	0.5	0	0.0	1.0	1.3	0	0.0
**Age (years)**								
*18 to 35*	20	9.9	7	9.2	13	16.5	0	0.0
*36 to 50*	66	32.7	30	39.5	26	32.9	10	21.3
*51 to 65*	76	37.6	23	30.3	27	34.2	26	55.3
*65 plus*	40	19.8	16	21.1	13	16.5	1	23.4
**State and territories**								
*New South Wales*	65	32.3	26	34.2	30	36.1	22	42.3
*Victoria*	31	15.4	9	11.8	14	17.9	8	17.0
*Queensland*	64	31.8	28	36.3	29	37.2	3	10.9
*Other states*	41	20.4	13	17.1	17	21.8	11	23.4
**Practice Setting**								
*Solo private practice*	137	67.8	51	67.1	51	64.6	35	74.5
*Group practice*	65	32.2	25	32.9	28	35.4	12	25.5
**Years of practice**								
*Less than 10 years*	51	25.2	19	25.0	23	29.1	9	19.1
*11 to 20*	72	35.6	27	35.5	31	39.2	14	29.8
*21 to 30*	48	23.8	19	27.7	16	20.3	13	27.7
*31 plus*	31	15.3	11	14.5	9	11.4	11	23.4

### Psychologists recommending types of CM products and/or practice and self-rated knowledge

Of the 201 psychologist responses included in the analysis, 5% (*n* = 11) reported having not recommended any type of CM, 32% (*n* = 65) reported having recommended one to three types, and 63% (*n* = 126) reported having recommended four or more types of CM products and/or practices (see Appendix Table 2). The rate ratios of psychologist response to statements about efficacy, risk, relevance and knowledge of CM with recommending and referring to a number of types of CM are reported in Table 3. The lowest rates of CM product and practice recommendations were from psychologists who agreed “CM is not scientifically valid” (RR = 0.77 [0.65; 0.93]), and that “referring to CM practitioners puts client safety at risk” (RR = 0.76 [0.63; 0.91. Of the 23% (*n* = 47) of participating psychologists who agreed with the statement that “CM as not scientifically valid”, some (*n* = 6) did not recommend any types of CM. However, most of those psychologists who agreed with this statement still recommended CM to their clients (*n* = 41).

**Table 2:** Demographic and practice characteristics of psychologists and recommending and referring to a number of types of CM

	Recommending CM products and practices			*p*	Referring to CM practitioners			*p*
	RR	Lower confidence limit	Upper confidence limit		RR	Lower confidence limit	Upper confidence limit	
**Gender **^a^				0.76				0.63
*Male*	ref				ref			
*Female*	0.97	0.81	1.17		0.94	0.75	1.18	
**Age (years)**				0.62				0.03
*18 to 35*	ref				ref			
*36 to 50*	0.96	0.74	1.24		1.34	0.92	1.95	
*51 to 65*	1.06	0.82	1.36		1.65	1.14	2.38	
*65 plus*	0.95	0.72	1.26		1.42	0.95	2.11	
**State and territories**				0.93				0.98
*New South Wales*	ref				ref			
*Victoria*	0.94	0.75	1.18		0.97	0.73	1.28	
*Queensland*	1.01	0.84	1.21		0.99	0.79	1.24	
*Other states*	1.00	0.82	1.24		1.03	0.80	1.32	
**Practice Setting**				0.79				0.18
*Solo private practice*	ref				ref			
*Group practice*	0.97	0.84	1.14		0.87	0.72	1.06	
**Years of practice**				0.37				0.004
*Less than 10 years*	ref				ref			
*11 to 20*	1.18	0.98	1.43		1.14	1.10	1.80	
*21 to 30*	1.12	0.91	1.38		1.19	0.91	1.58	
*31 plus*	1.14	0.90	1.44		1.63	1.22	2.16	
**AoPE/Specialty**				0.74				< 0.001
*General*	ref				ref			
*Clinical*	0.93	0.78	1.11		0.85	0.69	1.06	
*Other*	0.98	0.82	1.18		1.31	1.05	1.63	

^a^As there were not enough people in the gender category of *Other* to conduct a regression analysis they were not included

**Table 3:** Psychologist response to statements about efficacy, risk, relevance and knowledge of CM with recommending and referring to a number of types of CM

	Recommending CM products and practices			*p*	Referring to CM practitioners			*p*
	Rate ratio	Lower confidence limit	Upper confidence limit		Rate ratio	Lower confidence limit	Upper confidence limit	
**Agreement with statements about CM efficacy**								
CM is not scientifically valid	0.77	0.65	0.93	0.007	0.53	0.41	0.68	< 0.001
CM is not a good match with psychology	0.76	0.58	0.99	0.03	0.48	0.33	0.73	< 0.001
**Agreement with perspectives about risk and relevance of CM to psychology**								
CM treatments are unlikely to help those who use them as part of their mental health treatment	0.85	0.70	1.05	0.14	0.61	0.47	0.84	0.002
Current psychology ethical practice guidelines are adequate in guiding psychologists on how they can engage with their client’s CM use	0.96	0.82	1.12	0.63	0.81	0.66	0.99	0.04
It would be helpful if there were specific guidelines/policy related to psychology	1.01	0.79	1.28	0.93	1.37	0.97	1.92	0.07
Psychology as a field (including professional associations, academia, research) should provide more training on CM	1.35	1.06	1.71	0.01	2.10	1.47	2.99	< 0.001
Psychology as a field (including professional associations, academia, research) should provide more research on CM	1.35	1.07	1.70	0.01	1.93	1.38	2.71	< 0.001
Psychology as a field (including professional associations, academia, research) should provide more guidelines on CM	1.35	1.06	1.71	0.01	1.75	1.26	2.43	< 0.001
It is important for psychologists to understand and engage with their client’s preference for CM as part of their mental health treatment	1.36	0.97	1.90	0.68	4.08	2.03	8.21	< 0.001
There is potential to improve mental health outcomes with the integration of evidence-based CM within psychology practice	1.45	1.06	1.98	0.02	4.06	2.17	7.60	< 0.001
CM practitioners (e.g., naturopaths) can play a valuable role in assisting clients with their mental health problems	1.55	1.24	1.94	< 0.001	3.17	2.16	4.64	< 0.001
Psychologists should have knowledge of CM	1.35	1.08	1.68	0.008	1.68	1.24	2.27	< 0.001
Psychologists should learn about CM as part of their tertiary training	1.35	1.08	1.68	0.008	1.68	1.24	2.27	< 0.001
Psychology integrating with CM puts psychology’s reputation at risk	0.77	0.66	0.91	0.002	0.42	0.34	0.54	< 0.001
Referring clients to CM practitioners or services puts client safety at risk	0.76	0.63	0.91	0.003	0.39	0.29	0.52	< 0.001
**Self-rated knowledge of CM types as excellent/good**								
Aboriginal and Torres Strait Islander Traditional Medicine /Healing practices	1.35	1.03	1.76	0.03	1.71	1.26	2.31	< 0.001
Acupuncture	1.22	1.03	1.42	0.02	1.49	1.22	1.83	< 0.001
Dietary intervention	1.36	1.16	1.60	< 0.001	1.77	1.43	2.19	< 0.001
Exercise/movement interventions	1.18	1.02	1.37	0.02	1.26	1.05	1.52	0.01
Herbal medicine	1.26	1.07	1.48	0.005	1.54	1.27	1.87	< 0.001
Hypnotherapy	1.19	1.03	1.37	0.02	1.48	1.24	1.77	< 0.001
Massage	1.25	1.09	1.45	0.002	1.41	1.18	1.68	< 0.001
Meditation	1.51	1.15	1.96	0.003	1.55	1.11	2.16	0.01
Nutrition supplements	1.25	1.08	1.44	0.002	1.39	1.16	1.67	< 0.001
Probiotic supplements	1.28	1.10	1.49	0.001	1.28	1.06	1.55	0.009
Yoga	1.25	1.07	1.46	0.004	1.31	1.08	1.60	0.006

In contrast, psychologists who agreed with statements describing CM training and integration within psychology as beneficial, had the highest rates of recommending CM. For example, psychologists who agreed “CM practitioners can play a valuable role in assisting clients with their mental health problems” were more likely to recommend CM (RR = 1.55 [1.24; 1.94]).

The following CM types had the highest proportion of self-ratings as excellent/good among participating psychologists; meditation 88% (*n* = 178), dietary interventions 65% (*n* = 131), yoga 64% (*n* = 129), and exercise/movement interventions 54% (*n* = 110). With regards to the remaining seven CM types, more than half of the psychologists self-rated their knowledge as fair/poor for each type. Aboriginal and Torres Strait Islander Traditional Medicine /Healing practices attracted the lowest self-rated knowledge amongst the psychologists with only 6% (*n* = 12) of participants rating their knowledge as excellent/good for this medicine/healing practices. Psychologists who self-rated their knowledge of meditation as excellent/good had the highest rate of recommending multiple types of CM (RR = 1.51 [1.15; 1.96]).

### Psychologists referring to types of CM practitioners

Of the participants, 25% (*n* = 50) had not referred to any type of CM practitioner, 42% (*n* = 84) had referred to one to three types of CM practitioner, and 33% (*n* = 68) had referred to four or more types of CM practitioner. The lowest reported rates of referral to CM practitioners came from psychologists who agreed with the statement “referring clients to CM practitioners or services puts client safety at risk” (RR = 0.39 [0.29; 0.52). Psychologists who agreed with the statement “CM is not a good match with psychology” also reported low rates of referral to CM practitioners (RR = 0.48 [0.33; 0.73]). Of those psychologists who agreed with the statement “referring clients to CM practitioners or services puts client safety at risk” (*n* = 48), half (*n* = 24) reported referring to one or more types of CM practitioner. Meanwhile, another 10% (*n* = 21) of the study sample who also agreed with the statement “CM is not a good match with psychology”, half (*n* = 11) still reported referring to one or more CM practitioners. Across the demographic and practice characteristics psychologists aged 51 to 65, and those with 31 years plus experience, were more likely to refer to CM practitioners (Table 2). Clinical psychologists were the least likely to refer to any CM practitioner types. (See Appendix Table 1).

Psychologists who agreed with the statement “it is important for psychologists to understand and engage with their client’s preference for CM as part of their mental health treatment” had the highest reported rate of CM referral (RR = 4.08 [2.03; 8.21]). Similarly, psychologists who agreed with the statement “there is potential to improve mental health outcomes with the integration of evidence-based CM within psychology practice” also reported high rates of referral to CM practitioners (RR = 4.06 [2.17; 7.60). Similarly, psychologists who self-rated their knowledge of dietary intervention as excellent/good reported the highest rate of referring to multiple types of CM practitioner (RR = 1.77 [1.43; 2.19]).

## Discussion

This is the first study to examine the perspectives of Australian psychologists on CM as part of their clinical practice, and how this relates to the number of a range of CM products and/or practices they utilise and/or a range of CM practitioners they refer to as part of their clinical practice. One important finding from our study is that some psychologists appear to be engaging with CM products and/or practices, and CM practitioners even in those cases where the psychologist reports not perceiving CM as valid or efficacious. This finding—which contrasts with the closely held principle of evidence-based practice (EBP) that psychologists select empirically supported interventions [6, 53]—is, nevertheless, consistent with international research reflecting the complex interaction between health practitioner perspectives about CM, their lack of knowledge of CM, and their recommendations and/or referral behaviour regarding CM [17, 31, 36]. For example, research examining general practitioners suggests CM may act as useful resource with which these health professionals defend their clinical autonomy from what they perceive to be the threat of evidence based medicine [2]. It may be that the psychologists in our study are engaged in a similar stance in relation to clinical autonomy and EBP. However, further research is required to fully examine the validity of such an interpretation.

This finding from our study—that some psychologists appear to be engaging with CM products and/or practices, and CM practitioners even in those cases where the psychologist reports not perceiving CM as valid or efficacious—also appears to potentially add weight to the argument that engagement with CM may well be substantial across the psychology field [26, 28, 29, 48]. Identifying CM engagement in those critical cases where it is reasonable to consider it less likely (i.e., amongst psychologists who do not see CM as valid or efficacious) suggests the phenomenon may be widespread across other sections of the profession. This finding also suggests CM engagement amongst these psychologists may reflect, in part at least, a response to patient-led demand; while not necessarily seeing CM as valid or efficacious, it may be that some of the psychologists in our sample are driven to engage with CM as a result of repeated client request. Indeed, to this last point, our study findings also show psychologists who perceive engagement with client preference for CM as important, report the highest rates of referral to multiple types of CM practitioners. It may be that these issues are driving the level of engagement with CM amongst our sample. Unfortunately, our analysis is limited in its ability to validate such explanations and future research is required to further explore and test such explanations for CM engagement amongst psychologists.

Our study shows that a psychologist’s self-rated knowledge of dietary interventions as excellent/good predicts an increased likelihood of them ever referring clients to one or more CM practitioner types. This finding supports the observation that the role of nutrition in mental health care has recently emerged as a paradigm shift [5, 37, 50] with psychologists perceiving diet as an important part of their mental health care offerings [8, 38]. It may well be that the client referrals to nutrition-related CM practitioners (e.g., nutritionist, naturopath) identified in our study fit within this wider trend. However, our study data does not allow us to directly test these connections and further research needs to explore these issues in more detail.

The current study highlights that psychology professional associations may need to provide further CM relevant training for psychologists, given both a high level of psychologist engagement with CM and that 87% of surveyed psychologists perceive psychology as a field should provide more training on CM. Our findings relating to these specific issues have some similarities to the findings from studies of student doctors in Australia, where it was highlighted that nutrition education, for example, may not be sufficient to support nutrition related competencies in medical training and subsequently in their clinical practice [25, 41]. Findings reported in previous literature, as well as those identified from the current study, highlights that psychologists perceive gaps in their knowledge about a number of CM approaches, including nutrition, and how to integrate them into practice [8, 13, 38]. For example, discussing potential herb-nutrient-drug interactions when psychologists are educated and aware of potential risks may improve client safety. These findings, that psychologist perceive a need for more education in relevant CM, have implications for the field of psychology, how it manages scope of practice for psychologists, the inclusion of CM in psychologist tertiary training, as well as the provision of CM relevant professional development activities for psychologists. All these areas require further attention and empirical investigation to help understand the current implications and prospects of CM engagement amongst psychologists in clinical practice.

The current study suggests psychologists hold generally positive attitudes toward CM, despite limited knowledge of CM, a finding consistent with insights from previous research from Australia and abroad [17, 28]. This finding is important. Having limited knowledge of CM, yet recommending and referring to CM practitioners, may be problematic for psychologists in the context of contingent liability if they are unable to adequately explain or justify their referral to a CM practitioner. Perhaps psychologists do not see their self-rated knowledge of CM (as fair/poor) to be a barrier to referring their clients to CM practitioners. Perhaps at the interface of client preference for CM and their own, possibly conflicting, perspectives about efficacy, risk, relevance and self-rated knowledge of CM, psychologists prioritise the client’s preference for CM. Adding to psychologist’s dilemma regarding CM within clinical practice are other findings from the current study, that substantial numbers of psychologists perceive their knowledge of some CM types as fair/poor. This is consistent with Australian and international research that identifies limited guidelines and education for psychologists wishing to engage with CM in clinical practice in a number of jurisdictions [24, 27, 34, 36, 38, 51]. Again, further research is required to tease out and more deeply explore these and competing understandings and explanations around psychologists’ CM engagement.

Based on the findings from the current study, CM recommendation and CM practitioner referral are reported amongst those psychologists who perceive risks related to CM. It is unclear how psychologists reconcile and justify their risk perceptions regarding CM alongside their active recommendation of CM and referral to CM practitioners and this is an important area that requires further in-depth empirical enquiry. Within the framework of EBP, psychologists are advised to use the best research evidence in conjunction with clinical expertise and clients’ values, culture and preferences to inform mental health care [49]. It is unclear how psychologists interpret risks and evidence in CM without relevant CM guidelines, knowledge and competencies, as these are the devices through which evidence is supposedly interpreted into clinical practice. Further research may identify what motivates psychologists to engage with CM in clinical practice and how they reconcile limited relevant guidelines and related gaps in their knowledge.

### Limitations

This study is the first to focus on the rates of recommending and referring to types of CM amongst a diverse range of psychologists in clinical practice (e.g., in terms of years of experience, AoPE) in the context of psychologist perceptions of the risks and relevance of CM, and their self-rated knowledge of some types of CM. Although the number of participants in the study was small, it is representative of the Australian psychology workforce according to current workforce demographics provided by the Psychology Board of Australia (2022). Although the study participants were from some AoPE, not all psychology AoPEs were represented. Further, when interpreting the results, it is important to be mindful that most participants were either psychologists with general registration or psychologists with an AoPE in clinical psychology. We suggest caution when interpreting the results as there is potential for bias in our research due to participants being self-selecting. Those who have strong opinions on the relationship between psychology and CM are potentially more likely to respond.

## Conclusion

There are risks associated with psychologists engaging with CM in clinical practice when psychologists also perceive their self-rated knowledge to be poor. The findings from this study provide insights into psychologist perceptions of CM within psychology practice and how these perceptions are associated with rates of recommending and referring to CM as part of their clinical practice. These findings may well inform the development of CM relevant education and guidelines for psychologists. Further research is needed to determine what motivates psychologists to engage with CM (via recommendation or referral) in their clinical practice, how they justify such engagement and how they facilitate and accommodate such engagement for their clients.

### Supplementary Information

**Additional file 1:** **Table S1.** Psychologist demographic and practice characteristics and recommending or referring to CM

**Additional file 2:** **Table S2.** Statements about engagement with CM and recommending or referring to CM

**Additional file 3.** BMC CMT Data Sharing_How to access data

## Data Availability

The data that support the findings of this study are available

## Abbreviations

AAPI: Australian association of psychologists incorporated
AoPE: Area of practice endorsement
APS: Australian psychological society
CI: Confidence interval
CM: Complementary medicine
CHERRIES: Checklist for reporting results of internet e-surveys
EBP: Evidence-based practice
SPSS: Statistical package for the social sciences
