# Supplementary material for: Practice recommendations and referrals, perceptions of efficacy and risk, and self-rated knowledge regarding complementary medicine: a survey of Australian psychologists
Source: BMC Complement Med Ther. 2024 Jan 2;24:13. doi: 10.1186/s12906-023-04288-y (PMC10759583; doi:10.1186/s12906-023-04288-y)
Supplement: Supplementary file 1 — Additional file 1: Table S1. Psychologist demographic and practice characteristics and recommending or referring to CM [file 12906_2023_4288_MOESM1_ESM.docx]

APPENDIX Table 1. Psychologist demographic and practice characteristics and recommending or referring to CM

|  |  | Recommending CM products and practices | | | Referring to CM practitioners | | |
| --- | --- | --- | --- | --- | --- | --- | --- |
|  |  | Recommended none  (*n*=201) | Recommended 1 to 3 types  (*n*=200) | Recommended 4 plus types  (*n*=200) | Referred to none  (*n*=201) | Referred to 1 to 3 types  (*n*=200) | Referred to 4 plus types  (*n*=200) |
|  |  | *n* (%) | *n* (%) | *n* (%) | *n* (%) | *n* (%) | *n* (%) |
| **Gender** | |  |  |  |  |  |  |
|  | *Female* | 10 (90.9) | 53 (81.5) | 102 (81.0) | 44 (88.0) | 62 (73.8) | 59 (86.8) |
|  | *Male* | 1 (9.1) | 12 (18.5) | 23 (18.3) | 5 (10.0) | 22 (26.2) | 9 (13.2) |
|  | *Other* | 0 (0.0) | 0 (0.0) | 1 (0.8) | 1 (2.0) | 0 (0.0) | 0 (0.0) |
| **Age (years)** | |  |  |  |  |  |  |
|  | *18 to 35* | 1 (9.1) | 3 (4.6) | 16 (12.7) | 8 (16.0) | 8 (9.5) | 4 (5.9) |
|  | *36 to 50* | 3 (27.3) | 26 (40.0) | 37 (29.4) | 18 (36.0) | 29 (34.5) | 19 (27.9) |
|  | *51 to 65* | 4 (36.4) | 23 (35.4) | 49 (38.9) | 13 (26.0) | 32 (38.1) | 31 (45.6) |
|  | *65 plus* | 3 (27.3) | 13 (20.0) | 24 (19.0) | 11 (22.0) | 15 (17.9) | 14 (20.6) |
| **State and territories** | |  |  |  |  |  |  |
|  | *New South Wales* | 3 (27.3) | 22 (33.8) | 40 (32.0) | 14 (28.6) | 30 (35.7) | 21 (30.9) |
|  | *Victoria* | 4 (36.4) | 9 (13.8) | 18 (14.4) | 9 (18.4) | 11 (13.1) | 11 (16.2) |
|  | *Queensland* | 2 (18.2) | 22 (33.8) | 40 (32.0) | 18 (36.7) | 22 (26.2) | 24 (35.3) |
|  | *Other states* | 2 (18.2) | 12 (18.5) | 27 (21.6) | 8 (16.3) | 21 (25.0) | 12 (17.6) |
| **Practice Setting** |  |  |  |  |  |  |  |
|  | *Solo private practice* | 8 (72.7) | 43 (66.2) | 86 (68.3) | 32 (64.0) | 55 (65.5) | 50 (73.5) |
|  | *Group practice* | 3 (27.3) | 22 (33.8) | 40 (31.7) | 18 (36.0) | 29 (34.5) | 18 (26.5) |
| **Years of practice** | |  |  |  |  |  |  |
|  | *Less than 10 years* | 3 (27.3) | 21 (32.3) | 27 (21.4) | 18 (36.0) | 21 (25.0) | 12 (17.6) |
|  | *11 to 20* | 2 (18.2) | 22 (33.8) | 48 (38.1) | 14 (28.0) | 32 (38.1) | 26 (38.2) |
|  | *21 to 30* | 3 (27.3) | 15 (23.1) | 30 (23.8) | 13 (26.0) | 20 (23.8) | 15 (22.1) |
|  | *31 plus* | 3 (27.3) | 7 (10.8) | 21 (16.7) | 5 (10.0) | 11 (13.1) | 15 (22.1) |
| **AoPE^1^/Specialty** |  |  |  |  |  |  |  |
|  | *General* | 4 (63.4) | 23 (35.4) | 49 (38.9) | 23 (46.0) | 27 (32.1) | 26 (38.2) |
|  | *Clinical* | 3 (27.3) | 27 (41.5) | 49 (38.9) | 23 (46.0) | 38 (45.2) | 18 (26.5) |
|  | *Other* | 4 (36.4) | 15 (23.1) | 28 (22.2) | 4 (8.0) | 19 (22.6) | 24 (35.3) |

^1^Area of Practice Endorsement
