# Supplementary material for: Practice recommendations and referrals, perceptions of efficacy and risk, and self-rated knowledge regarding complementary medicine: a survey of Australian psychologists
Source: BMC Complement Med Ther. 2024 Jan 2;24:13. doi: 10.1186/s12906-023-04288-y (PMC10759583; doi:10.1186/s12906-023-04288-y)
Supplement: Supplementary file 2 — Additional file 2: Table S2. Statements about engagement with CM and recommending or referring to CM [file 12906_2023_4288_MOESM2_ESM.docx]

APPENDIX Table 2. Statements about engagement with CM and recommending or referring to CM

|  | Recommending CM products and practices | | | Referring to CM practitioners | | |
| --- | --- | --- | --- | --- | --- | --- |
|  | Recommended none  (*n*=201) | Recommended 1 to 3 types  (*n*=200) | Recommended 4 plus types  (*n*=200) | Referred to none  (n=201) | Referred to 1 to 3 types  (*n*=200) | Referred to 4 plus types  (*n*=200) |
|  | *n* (%) | *n* (%) | *n* (%) | *n* (%) | *n* (%) | *n* (%) |
| **Agreement with statements about CM efficacy** |  |  |  |  |  |  |
| CM is not scientifically valid | 6 (66.7) | 20 (30.8) | 21 (16.7) | 17 (35.4) | 25 (29.8) | 5 (7.4) |
| CM is not a good match with psychology | 2 (22.2) | 12 (18.5) | 7 (5.6) | 10 (20.8) | 9 (10.7) | 2 (2.9) |
|  | | | | | | |
| **Agreement with perspectives about risk and relevance of CM to psychology** |  |  |  |  |  |  |
| CM treatments are unlikely to help those who use them as part of their mental health treatment | 2 (20.0) | 16 (25.0) | 15 (11.9) | 13 (26.5) | 12 (14.5) | 8 (11.8) |
| Current psychology ethical practice guidelines are adequate in guiding psychologists on how they can engage with their client’s CM use | 3 (30.0) | 21 (32.3) | 36 (28.6) | 19 (38.8) | 25 (29.8) | 16 (23.5) |
| It would be helpful if there were specific guidelines/policy related to psychology | 8 (88.9) | 58 (89.2) | 114 (90.5) | 38 (79.2) | 80 (95.2) | 62 (91.2) |
| Psychology as a field (including professional associations, academia, research) should provide more training on CM | 5 (50.0) | 55 (84.6) | 114 (90.5) | 32 (65.3) | 79 (94.0) | 63 (92.6) |
| Psychology as a field (including professional associations, academia, research) should provide more research on CM | 6 (60.0) | 51 (78.5) | 116 (92.1) | 33 (67.3) | 76 (90.5) | 64 (94.1) |
| Psychology as a field (including professional associations, academia, research) should provide more guidelines on CM | 7 (70.0) | 49 (75.4) | 118 (93.7) | 35 (71.4) | 76 (90.5) | 63 (92.6) |
| It is important for psychologists to understand and engage with their client’s preference for CM as part of their mental health treatment | 9 (90.0) | 57 (87.7) | 122 (96.8) | 40 (81.6) | 80 (95.2) | 68 (100.0) |
| There is potential to improve mental health outcomes with the integration of evidence-based CM within psychology practice | 7 (70.0) | 58 (89.2) | 120 (95.2) | 39 (79.6) | 79 (94.0) | 67 (98.5) |
| CM practitioners (e.g., naturopaths) can play a valuable role in assisting clients with their mental health problems | 6 (60.0) | 46 (70.8) | 116 (92.1) | 31 (63.3) | 70 (83.3) | 67 (98.5) |
| Psychologists should have knowledge of CM | 7 (70.0) | 48 (73.8) | 115 (91.3) | 34 (69.4) | 74 (88.1) | 62 (91.2) |
| Psychologists should learn about CM as part of their tertiary training | 7 (70.0) | 48 (73.8) | 115 (91.3) | 34 (69.4) | 74 (88.1) | 62 (91.2) |
| Psychology integrating with CM puts psychology’s reputation at risk | 7 (70.0) | 30 (46.2) | 30 (23.8) | 30 (61.2) | 30 (35.7) | 7 (10.3) |
| Referring clients to CM practitioners or services puts client safety at risk | 5 (55.6) | 23 (35.4) | 20 (15.9) | 24 (50.0) | 19 (22.6) | 5 (7.4) |
|  | | | | | | |
| **Self-rated knowledge of CM types as excellent/good** |  |  |  |  |  |  |
| Aboriginal and Torres Strait Islander Traditional Medicine /Healing practices | 0 (0.0) | 0 (0.0) | 12 (9.5) | 0 (0.0) | 4 (4.8) | 8 (11.8) |
| Acupuncture | 1 (10.0) | 7 (10.8) | 31 (24.6) | 4 (8.2) | 15 (17.9) | 20 (29.4) |
| Dietary intervention | 4 (40.0) | 28 (43.1) | 99 (78.6) | 23 (46.9) | 52 (61.9) | 56 (82.4) |
| Exercise/movement interventions | 4 (40.0) | 27 (41.5) | 79 (62.7) | 18 (36.7) | 51 (60.7) | 41 (60.3) |
| Herbal medicine | 1 (10.0) | 8 (12.3) | 35 (27.8) | 6 (12.2) | 14 (16.7) | 24 (35.3) |
| Hypnotherapy | 4 (40.0) | 16 (24.6) | 53 (42.1) | 13 (26.5) | 22 (26.2) | 38 (55.9) |
| Massage | 3 (30.0) | 19 (29.2) | 68 (54.0) | 17 (34.7) | 34 (40.5) | 39 (57.4) |
| Meditation | 7 (70.0) | 53 (81.5) | 118 (93.7) | 40 (81.6) | 74 (88.1) | 64 (94.1) |
| Nutrition supplements | 3 (30.0) | 16 (24.6) | 62 (49.2) | 14 (28.6) | 33 (39.3) | 34 (50.0) |
| Probiotic supplements | 1 (10.0) | 9 (13.8) | 47 (37.3) | 12 (24.5) | 21 (25.0) | 24 (35.3) |
| Yoga | 5 (50.0) | 33 (50.8) | 91 (72.2) | 28 (57.1) | 51 (60.7) | 50 (73.5) |

*Note*. In the table *n* refers to the number of participants who recommended or referred as per the amount stated in the column heading. The % refers to the percentage of participants who recommended or referred as per the amount stated in the column heading.
