# Supplementary material for: Practice recommendations and referrals, perceptions of efficacy and risk, and self-rated knowledge regarding complementary medicine: a survey of Australian psychologists
Source: BMC Complement Med Ther. 2024 Jan 2;24:13. doi: 10.1186/s12906-023-04288-y (PMC10759583; doi:10.1186/s12906-023-04288-y)
Supplement: Supplementary file 3 — Additional file 3. BMC CMT Data Sharing_How to access data [file 12906_2023_4288_MOESM3_ESM.docx]

The datasets generated and/or analysed during the current study are available in the University of Technology Sydney repository (DOI: 10.26195/ecvr-vq16).
